# Multicomponent Macrocyclic IL-17a Modifier

**DOI:** 10.1021/acsmedchemlett.2c00257

**Published:** 2022-08-12

**Authors:** Eman Abdelraheem, Max Lubberink, Wenja Wang, Jingyao Li, Atilio Reyes Romero, Robin van der Straat, Xiaochen Du, Matthew Groves, Alexander Dömling

**Affiliations:** † Department of Pharmacy, Drug Design Group, University of Groningen, A. Deusinglaan 1, Groningen 9700 AV, The Netherlands; § Chemistry Department, Faculty of Science, Sohag University, Sohag 82524, Egypt

**Keywords:** interleukin 17a, IL-17a, multicomponent reaction, Ugi, isocyanide, MST, pharmacophore

## Abstract

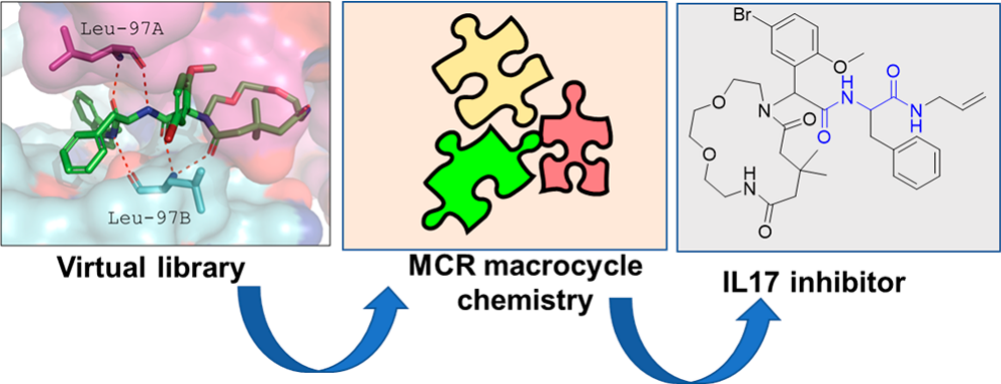

IL-17a is a major inflammation target, with several approved antibodies in clinical use. Small-molecule IL-17a antagonists are an emerging hot topic, with the recent advancement of three compounds into clinical trials. Here, we describe the design, discovery, synthesis, and screening of macrocyclic compounds that bind to IL-17a. We found that all currently described IL-17a modifiers belong to the same pharmacophore model, likely resulting in a similar receptor binding mode on IL-17a. A pipeline of pharmacophore analysis, virtual screening, resynthesis, and protein biophysics resulted in a potent IL-17a macrocyclic modifier.

The cytokine interleukin 17a (IL-17a) is involved in pathogenesis of several immunoinflammatory diseases, including psoriasis, psoriatic arthritis, and rheumatoid arthritis. After binding to the receptor on the surface of T helper cells, IL-17 activates several signaling cascades that, in turn, lead to the induction of chemokines. The chemokines act as chemoattractants and recruit immune cells, such as monocytes and neutrophils, to the site of inflammation to help eliminate invading pathogens. Activation of IL-17 signaling is also observed in the pathogenesis of various autoimmune disorders, such as psoriasis. Antagonizing the IL-17–receptor interaction can abrogate the inflammatory overreaction of this cytokine in a pathogenic setting.^1^ The potential applications of IL17-directed drugs could go well beyond the above-mentioned indications, e.g., multiple sclerosis (MS), Alzheimer’s disease, or ischemic brain injury; however, they are limited by the mAb nature of the currently available drugs.^2^

With an extensive buried surface area between the receptor and the IL-17 dimer of ∼2220 Å^2^, not surprisingly, all current anti-IL-17a therapies are based on antibodies. Given the success of the marketed antibody drugs secukinumab (Cosentyx) and ixekizumab (Talz) in psoriasis, psoriatic arthritis, and ankylosing spondylitis, the race to a commercial small-molecule IL-17a antagonist has started (Figure 1). While the early attempts to discover IL-17a modifiers were focused on macrocycles, with the idea to cover a large surface area (**1**–**4**),^3−6^ more recent attempts successfully discovered non-macrocyclic small molecules (**5**–**7**).^7,8^ It started as early as 2016, when screening a macrocyclic DEL-derived library yielded the 18-membered compound **1**.^3^ Several years later, the co-crystal structures of related macrocycles **2** (20-membered) and **3** (21-membered), with the IL-17a homodimer, were published, revealing that these compounds bind at the interface of the monomers of the IL-17a dimer, thereby allosterically reducing its ability to engage the IL-17 receptor.^4,5^ This small-molecule mode-of-action distortion of a dimeric or oligomeric protein ligand is also observed in other chemokines, e.g., the anti-tumor necrosis factor TNF-α.^9^ Another interesting IL-17a-modifying macrocycle is the 18-membered **4**, which is built on a macrolide scaffold. Recently, three small non-macrocyclic molecules, **5**, **6**, and **7**, entered early clinical trials.^10−12^ In parallel, several peptides potently binding IL-17a were disclosed.^4,13,14^ Here we describe our efforts to discover yet another macrocyclic IL-17a modifier, based on our recently developed efficient multi-component reaction-based macrocycle chemistry.^15−22^

**Figure 1:**
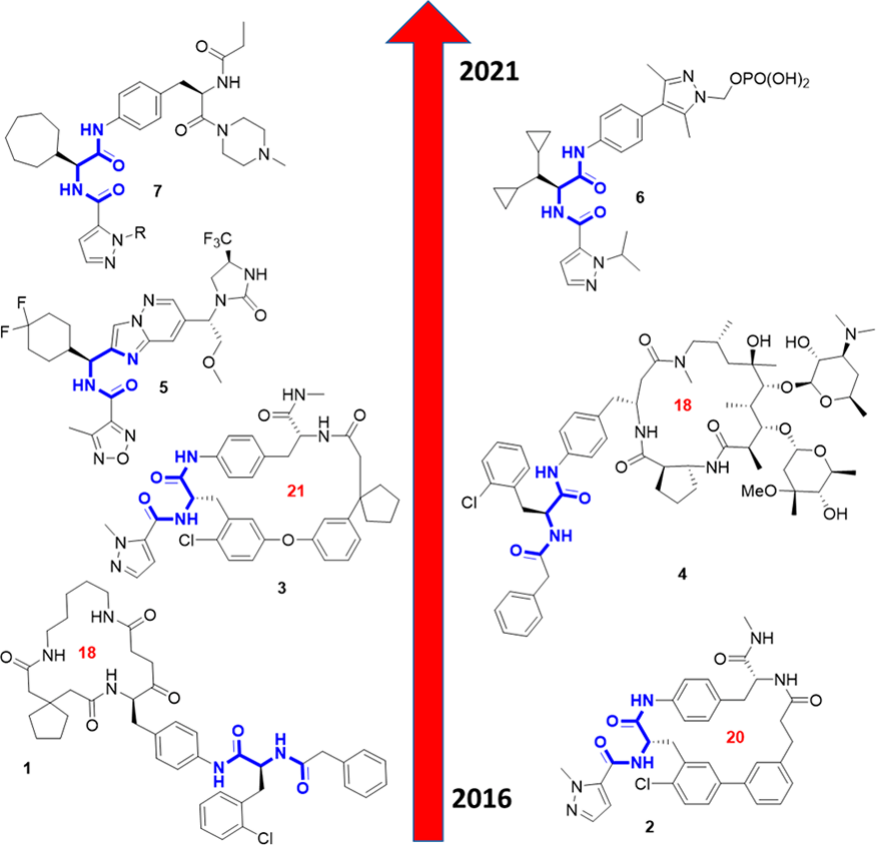
Timeline of macrocyclic and small-molecule IL-17a modifier discovery. The smallest common denominator α-aminoacyl amide substructure is marked in blue.

Analysis of the co-crystal structure of the two macrocycles, **2** and **3**, bound to the interface of the IL-17a dimer revealed in both structures four key hydrogen bonds of the bis-amide substructure with the backbone of Leu97A and Leu97B (Figure 2B).^23^ We figured that these hydrogen bonds could be used to anchor moieties into the IL-17a dimer interface.

**Figure 2:**
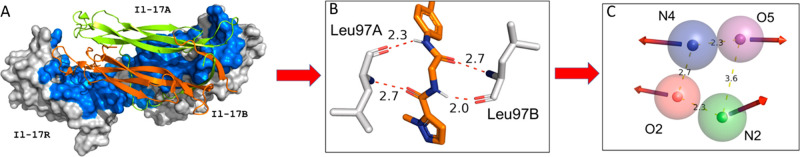
Common pharmacophore model derived from IL-17a modifier and co-crystal structures. (A) IL-17a dimer (gold and green cartoon) bound to IL-17 receptor (gray surface; buried surface shown in blue, PDB ID 4HSA). (B) Zoom into the key hydrogen bond donor/acceptor feature of the macrocycle **3** bound to IL-17a dimer Leu97A/B (PDB ID 5HI4). (C) Pharmacophore model featuring the two hydrogen bond donors and acceptors found in the bis-amide substructure.

Interestingly, all currently described IL-17a modifiers (Figure 1) contain the same bis-amide substructure or a bioisostere thereof (**5**). Therefore, it is conceivable that they all bind to similar sites on IL-17a. Indeed, all the structures shown in Figure 1 can be convincingly modeled onto the published co-crystal structure of IL-17a and the macrocycle.^5^ Our bis-hydrogen bond donor/hydrogen bond acceptor pharmacophore model (Figure 2C) served to screen a compound library of ∼1000 macrocycles by virtual screening (VS). To test our VS hypothesis in a timely manner, we focused on a recently described (by us) short and convergent two-step macrocycle synthesis (Scheme 1). For this, we created a virtual library of 1000 macrocycles.^24^ The chemistry consisted of a mono-acylation of an α,ω-bis primary amine with a cyclic carboxylic acid anhydride.^15^ The resulting α,ω-amino carboxylic acid was then subject to an intramolecular Ugi reaction, employing an isocyanide and an oxo component, to yield the highly functionalized macrocycle. To introduce the bis-amide functionality into the macrocyclic product, the methyl esters isocyanides were coupled to primary amines before the Ugi reaction was performed.^25^ Alternatively, the macrocyclic methyl ester Ugi product underwent aminolysis with amines after isolation of the macrocyclic methyl ester.

**Scheme 1:**
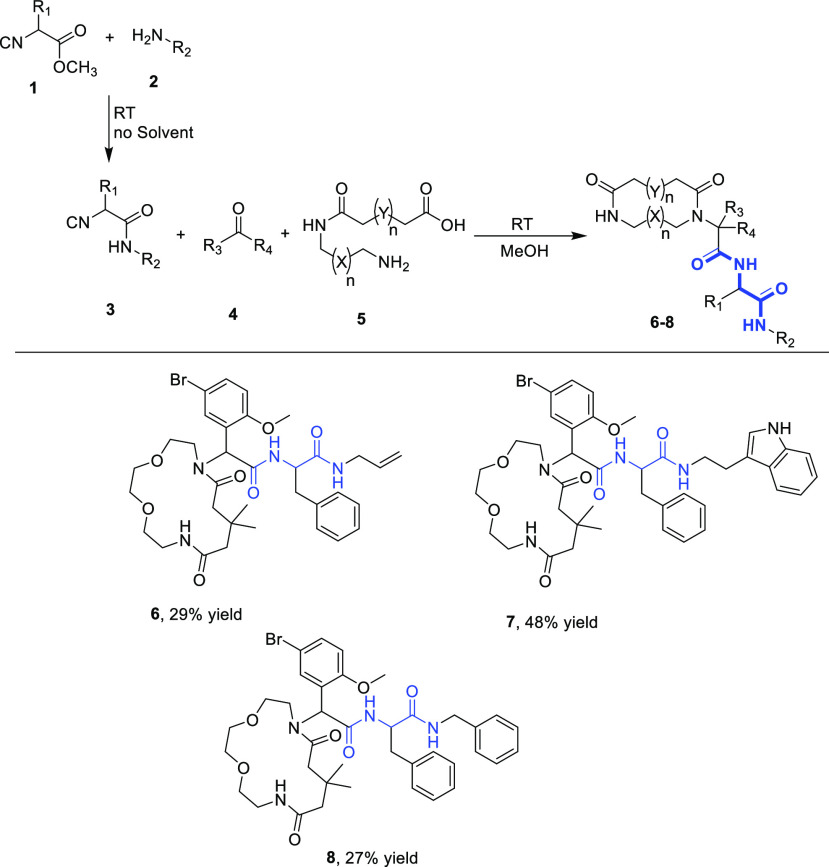
Two-Step Synthesis of Complex Macrocycles: Top Three Resynthesized Macrocycles (**6**–**8**) from a Virtual Library of 1000 Macrocycles

The resynthesis of the top VS hits (**6**–**8**) is shown in Scheme 1. The total yields of **6**, **7**, and **8** were 29%, 48%, and 27%, respectively. Next, we determined the binding affinity of the three compounds to the IL-17a dimer using microscale thermophoresis (MST). The mixture of stereoisomers of **6**, **7**, and **8** showed promising binding affinities of 507 nM, 94 μM, and 51.1 μM, respectively. To further characterize the most active compound, **6**, we separated the stereoisomers using semi-preparative supercritical fluid chromatography (SFC) on a Chiralpak IC chiral column, 10.0 × 250 mm (Figure 3). We were able to separate two isomers, **6C** and **6D**. Rescreening of the two isolated stereoisomers and the remaining inseparable mix revealed **6AB** and showed that the isomer **6C** gave the best affinity, at 170 nM.

**Figure 3:**
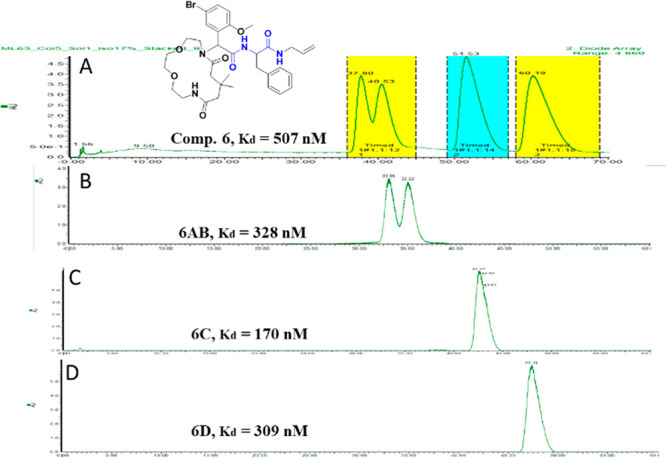
SFC-MS chromatograms of the separation of the stereoisomers of **6** on a Chiralpak IC chiral column and their respective binding affinities, *K*_d_: (A) (*rac*)-**6**, (B) **6AB**, (C) **6C**, and (D) **6D**.

Among the four possible stereoisomers of **6**, the *SS* one shows the best cooperativity score (14.6), followed by *RS* (12.8), *SR* (11.6), and *RR* (7.2). Figure 4A shows a dense network of van der Waals interactions between **6-SS** and hydrophobic residues like Ile-96, Leu-97, Val-98, Leu-99, Leu-112, and Leu-117. Similarly, these types of interactions are also prevalent among the other three stereoisomers (Figure SI-7). To a lesser but still present extent, there are π–π contacts with Tyr-62 and Leu-99 (Figure 4B). Interestingly, the four hydrogen bonds with Leu-97A/B are shared only by the *SS* stereoisomer in **7** and **8** (Figure 4C and Figure SI-7). In contrast, the other three stereoisomers do not display this pattern of key interactions. Nevertheless, **7** and **8** contact hydrophobic amino acids like **6**. When comparing the cooperativity binding scores, however, there are considerable differences among the macrocycles, as can be seen in Table SI-2. Van der Waals interactions outnumber the others across the seven types of interactions counted and therefore should be considered crucial for macrocycle binding to IL-17a.

**Figure 4:**
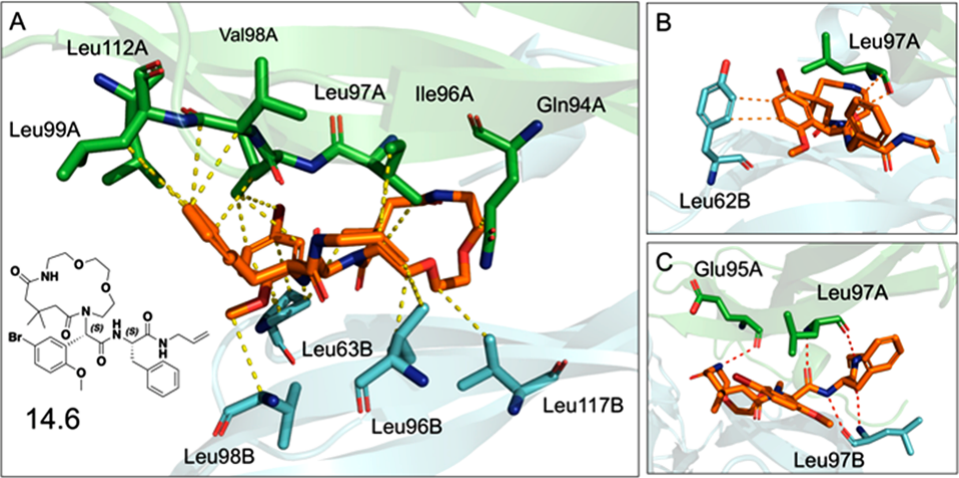
Docking poses of the **6-SS** stereoisomer in the cavity of IL-17a. The A and B chains of IL-17a are depicted as green and cyan ribbons, respectively. The same color pattern is applied to amino acids (stick representation) involved in molecular contacts with **6**: (A) van der Waals interactions (yellow dotted lines), (B) π–π interactions (orange dotted lines), and (C) hydrogen bonds (red dotted lines).

To determine the affinity of the compounds, an *in vitro* IL-17a MST assay was performed. Purified IL-17a protein was labeled with the monolith His-Tag labeling kit RED-tris-NTA. The compounds described in this application were tested for their ability to bind IL-17. The biophysical data obtained from testing the above representative examples **6**–**8** and the separated stereoisomers **6** using MST revealed binding affinities of 507 nM, 94 μM, and 51.1 μM for the diastereoisomeric *meso* mixture of compounds **6**, **7**, and **8**, respectively (Figure 3). Subsequent separation of compound **6** into **6AB**, **6C**, and **6D** revealed *K*_d_ values of 328 nM, 170 nM, and 309 nM, respectively.

Using a rational drug design approach, enabled by computational macrocycle screening and a very short, two-step macrocycle synthesis, we were able to discover low nM binders to the important anti-inflammatory target IL-17a. Moreover, pharmacophore analysis of currently described small-molecule IL-17a antagonists revealed a common multifurcated hydrogen-bonding pattern. Our findings are significant and will be of help for future design of small-molecule IL-17a antagonists to test medical indications which are out of reach of the current mAb-based therapeutics.

## Supporting Information Available

Experimental procedures and full characterization for compounds, including Figures SI-1–SI-8 and Tables SI-1 and SI-2 (PDF)
