# Supplementary material for: Multicomponent Macrocyclic IL-17a Modifier
Source: ACS Med Chem Lett. 2022 Aug 12;13(9):1468–71. doi: 10.1021/acsmedchemlett.2c00257 (PMC9465830; doi:10.1021/acsmedchemlett.2c00257)
Supplement: Supplementary file 1 — ml2c00257_si_001.pdf [file ml2c00257_si_001.pdf]

Supporting Information

Multicomponent Macrocyclic IL-17a Modifier

Eman Abdelraheem[†§], Max Lubberink[†], Wenja Wang[†], Jingyao Li[†], Atilio Reyes Romero[†], Robin van der Straat[†], Xiaochen Du[†], Matthew Groves[†], Alexander Dömling[†,*]

[†]Department of Pharmacy, Drug Design Group, University of Groningen, A. Deusinglaan 1, Groningen 9700AV, The Netherlands.

[§]Chemistry Department, Faculty of Science, Sohag University, Sohag 82524, Egypt

Table of Contents

General information	S3
General Experimental Procedures	S3-S4
SFC-MS separation for a diastereomeric mixture	S5-S12
Evaluation of inhibitory activity of compounds towards IL17	S13-S14
Docking procedure, library construction, graphs and pictures of pharmacophore	S15-S17
References	S18

Experimental section

General information:

Nuclear magnetic resonance spectra (NMR) were recorded on a Bruker Avance 500 spectrometer, Proton nuclear magnetic resonance spectra (^{1}H NMR (500 MHz), Carbon nuclear magnetic resonance spectra ^{13}C NMR (126 MHz)). Chemical shifts for ^{1}H NMR were reported as δ values and coupling constants were in hertz (Hz). The following abbreviations were used for spin multiplicity: s = singlet, d = doublet, t = triplet, dd = double doublet, m = multiplet, bs = broad singlet. Chemical shifts for ^{13}C NMR reported in ppm relative to the solvent peak. Thin layer chromatography was performed on Fluka precoated silica gel plates (0.20 mm thick, particle size 25 µm). Flash chromatography was performed on a Teledyne ISCO Combiflash Rf, using RediSep Rf Normal-phase Silica Flash Columns (Silica Gel 60 Å, 230 - 400 mesh). Reagents were available from commercial suppliers and used without any purification unless otherwise noted. All isocyanides were made in house. Other reagents were purchased from Sigma Aldrich, ABCR, Acros and AK Scientific and were used without further purification. Mass spectra were measured on a Waters Investigator Supercritical Fluid Chromatograph with a 3100 MS Detector (ESI) using a solvent system of methanol and CO_2 on a Viridis silica gel column (4.6 × 250 mm, 5 µm particle size) and reported as (m/z). Electrospray ionization mass spectra (ESI-MS) were recorded on a Waters Investigator Semi-prep 15 SFC-MS instrument. Yields given refer to chromatographically purified and spectroscopically pure compounds unless otherwise stated. No unexpected or unusually high safety hazards were encountered.

General Experimental Procedures:

Procedure A: General procedure for synthesis of α,ω-amino carboxylic acids: Diamine (1.0 mmol) was dissolved in THF (6 mL), then a solution of anhydride (1.0 mmol) in THF (4 mL) was added dropwise. The reaction mixture was stirred for 2h. The product precipitated, filtered off and dried under a high vacuum to afford the amino carboxylic acid product.

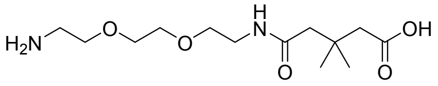

5-((2-(2-(2-aminoethoxy)ethoxy)ethyl)amino)-3,3-dimethyl-5-oxopentanoic acid[1] : The product was obtained as a colorless oil (77%); ^{1}H NMR (500 MHz, D_2O) δ 3.66 (t, J = 5.1 Hz, 2H), 3.61 (s, 4H), 3.58 (t, J = 4.8 Hz, 2H), 3.38 (t, J = 5.2 Hz, 2H), 3.12 (t, J = 5.3 Hz, 2H); 2.12 (s, 2H), 2.00 (s, 2H); 0.99 (s, 6H); ^{13}C NMR (126 MHz, D_2O) δ 177.0, 172.6, 70.1, 69.5, 69.5, 69.4, 68.7, 66.4, 39.1, 38.4, 30.5, 26.1.

Procedure B: General procedure for synthesis of isocyanide by the reaction of primary amines with substituted isocyanoacetic acid methyl ester [2]:

10 mmol of amine and 10 mmol of isocyanoacetic acid methyl ester was added together. The reaction mixture was stirred overnight at room temperature. In most cases the product precipitated during the reaction, it was filtered off, washed with cold diethyl ether, and dried under vacuum overnight. If the product did not precipitate, cold diethyl ether was added to the reaction mixture, and the product was allowed to crystallize in the freezer.

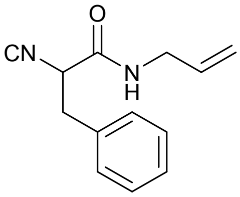

N-allyl-2-isocyano-3-phenylpropanamide: The product was obtained as a yellow oil (45%,); ^{1}H NMR (500 MHz, DMSO) δ 3.45 (dd, J = 6.2 Hz, 1H), 3.21 (dd, J = 7.4 Hz, J = 9.2 Hz, 1H), 3.72 (d, 2H), 4.79 (t, 1H), 5.21 (d, 1H), 5.25 (d, 1H), 5.91 (m, 1H), 7.43- 7.56 (m, 5H). ^{13}C NMR (DMSO, 126 MHz) δ 36.2, 42.8, 62.1, 117.1, 125.3, 127.5, 128.1, 128.2, 134.8, 139.2, 155.3, 175.3.

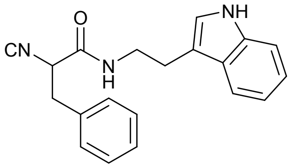

N-(2-(1H-indol-3-yl)ethyl)-2-isocyano-3-phenylpropanamide: The product was obtained as a white solid (60%,); ^{1}H NMR (500 MHz, DMSO) δ 2.91-2.97 (tr, 2H), 3.11-3.21 (m, 2H), 3.45 (tr, 2H), 4.55(tr, 1H), 6.97 (s, 1H), 7.01 (m, 1H), 7.21-7.32 (m, 5H),

7.41(d, 1H), 7.53 (d, 1H). ^{13}C NMR (DMSO, 126 MHz) δ 24.5, 39.1, 41.5, 60.1, 111.7, 111.8, 118.1, 118.9, 122.2, 122.4, 127.7, 129.4, 130.3, 135.8, 137.3, 155.1, 160.0, 166.5, 174.5.

N-benzyl-2-isocyano-3-phenylpropanamide: The product was obtained as a white solid (75%,); ^{1}H NMR (500 MHz, DMSO) δ 2.77 (dd, J = 6.2 Hz, 1H), 3.11 (dd, J = 6.1 Hz, J = 9.9 Hz, 1H), 3.99 (s, 2H), 4.23 (t, 1H), 6.34 (br, 1H), 7.11- 7.36 (m, 10H). ^{13}C NMR (DMSO, 126 MHz) δ 36.6, 42.3, 62.9, 127.2, 127.6, 128.4, 128.9, 129.2, 139.4, 139.7, 156.7, 173.3.

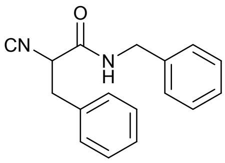

Procedure C1: General procedure for Ugi-macrocyclization: In 100 mL round bottom flask equipped with a magnetic stir bar, the α,ω-amino carboxylic acid (1.0 mmol, 1 equiv.) was dissolved in methanol (10.0 ml). Aldehyde (1.0 mmol, 1.0 equiv.) was added and the solution was stirred for 30 min at rt in order to do schiff base, then isocyanide (1.0 mmol, 1 equiv.) was added to the reaction mixture and stirred for 24 h. After completion of the reaction, as monitored by TLC and LC – MS, the reaction mixture was dried under reduced pressure via rotary evaporation and the residue was purified using flash chromatography (CH_2Cl_2: MeOH 9:1) to afford macrocycles.

Procedure C2: General procedure for macrocyclization and aminolysis of macrocyclic methyl ester Ugi-products[3] :

Step 1: In 100 mL round bottom flask equipped with a magnetic stir bar, the α,ω-amino carboxylic acid (1.0 mmol, 1 equiv.) was dissolved in methanol (10.0 ml). Aldehyde (1.0 mmol, 1.0 equiv.) was added and the solution was stirred for 30 min at rt in order to do schiff base, then isocyanide methyl ester for phenylalanine (1.0 mmol, 1 equiv.) was added to the reaction mixture and stirred for 24 h. After completion of the reaction, as monitored by TLC and LC – MS, the reaction mixture was dried under reduced pressure and the residue was purified using flash chromatography (CH_2Cl_2: MeOH 9:1) to afford macrocycles.

Step 2: To the stirred solution of macrocycle methyl ester (1.0 mmol, 1.0 equiv.) in THF (2 mL) was added TBD (30 mol %) then a solution of allylamine or benzylamine or tryptamine (1.0 mmol in 4mL THF) was added dropwise. The reaction mixture was slowly warmed to 75 °C and stirred for overnight. After completion of the reaction, as monitored by TLC, was allowed to cool to ambient temperature and concentrated in vacuo. The product was purified using flash chromatography using ethyl acetate/cyclohexane (2:8).

N-allyl-2-(2-(5-bromo-2-methoxyphenyl)-2-(10,10-dimethyl-8,12-dioxo-1,4-dioxa-7,13-diazacyclopentadecan-7-yl)acetamido)-3-phenylpropanamide 6:

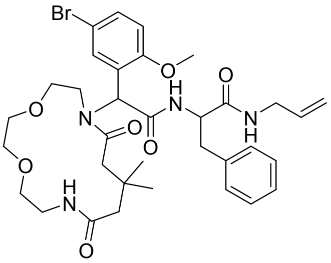

^{1}H NMR (500 MHz, Chloroform-d) δ 7.51 – 7.40 (m, 2H), 7.27 (d, J = 7.4 Hz, 1H), 7.24 – 7.16 (m, 3H), 7.00 (dd, J = 6.6, 3.4 Hz, 1H), 6.97 – 6.89 (m, 1H), 6.79 (dd, J = 17.9, 9.0 Hz, 1H), 6.56 (d, J = 8.0 Hz, 1H), 5.83 (ddt, J = 17.4, 10.6, 5.6 Hz, 2H), 5.55 (s, 1H), 5.32 – 4.99 (m, 3H), 4.67 (td, J = 8.2, 5.5 Hz, 1H), 3.90 (tt, J = 7.2, 1.6 Hz, 1H), 3.77 (d, J = 7.8 Hz, 3H), 3.62 – 3.45 (m, 6H), 3.46 – 3.19 (m, 8H), 2.61 – 2.44 (m, 4H), 1.12 – 1.06 (m, 6H). ^{13}C NMR (126 MHz, Chloroform-d) δ 176.1, 172.5, 170.9, 156.4, 134.0, 133.8, 132.9, 132.7, 129.1, 128.9, 128.6, 126.7, 125.8, 116.5, 113.4, 112.4, 69.6, 69.4, 69.3, 67.7, 61.4, 59.2, 55.9, 55.8, 47.1, 42.1, 41.9, 39.4, 36.1, 34.1, 33.9, 29.5.

N-(2-(1H-indol-3-yl)ethyl)-2-(2-(5-bromo-2-methoxyphenyl)-2-(10,10-dimethyl-8,12-dioxo-1,4-dioxa-7,13-diazacyclo pentadecan -7-yl)acetamido)-3-phenylpropanamide 7:

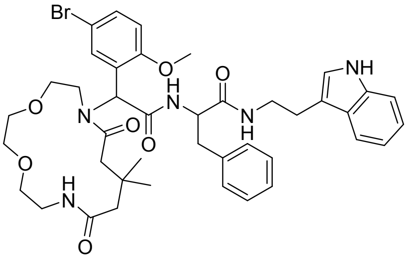

^{1}H NMR (500 MHz, Chloroform-d, 1:1 diastereomeric ratio) δ 8.39 (s, 1H), 8.31 (s, 1H), 7.60 (dd, 3H), 7.51 (s, 1H), 7.44 (d, J = 2.6 Hz, 1H), 7.43 – 7.39 (m, 3H), 7.36 – 7.21 (m, 9H), 7.19 – 7.14 (m, 2H), 7.10 (d, 1H), 6.8 (d, J = 7.4 Hz, 1H), 6.78 (d, J = 7.1 Hz, 1H), 6.73 (d, J = 8.4 Hz, 1H), 6.59 (s, 1H), 6.51 (d, J = 7.6 Hz, 2H), 5.51 (s, 1H), 5.30 (s, 1H), 4.54 – 4.51 (m, 1H), 3.59 (s, 3H), 3.51 (s, 3H), 3.49 – 3.42 (m, 11H), 3.41- 3.34 (m, 9H), 3.33 – 3.30 (m, 11H), 3.27 – 3.24 (m, 3H), 3.23 – 3.19 (m, 3H), 3.17 - 3.06 (m, 6H), 2.75 (d, 1H),

2.71- 2.66 (m, 2H), 2.57 - 2.55 (m, 2H), 2.54 – 2.51 (m, 2H), 2.47 (d, 1H), 1.22 (s, 3H), 1.06 (s, 3H),1.03 (s, 3H), 0.96 (s, 3H). ^{13}C NMR (126 MHz, CDCl3) δ 175.8, 175.7, 172.5, 172.3,170.9, 170.7, 169.4, 168.2, 156.5, 156.4, 137.6, 136.3, 136.2, 132.8, 132.6, 132.1, 129.1, 129.0, 128.6, 128.5, 128.3, 127.4, 127.3, 126.7, 126.6, 125.6, 125.4, 125.4 ,122.5, 122.2, 121.8, 121.7, 119.2, 119.1, 118.6, 113.2, 113.1, 112.9, 112.6, 112.4, 111.3, 111.2, 69.5, 69.4, 69.3, 69.2, 69.1, 67.7, 61.2, 59.2, 55.9, 55.8, 55.1, 46.9, 46.6, 41.8, 41.6, 40.0, 39.3, 39.1, 36.4, 36.1, 33.9, 33.7, 29.6, 29.4, 29.3, 25.4, 25.3, 25.2.

N-benzyl-2-(2-(5-bromo-2-methoxyphenyl)-2-(10,10-dimethyl-8,12-dioxo-1,4-dioxa-7,13-diazacyclopentadecan-7-yl)acetamido)-3-phenylpropanamide 8:

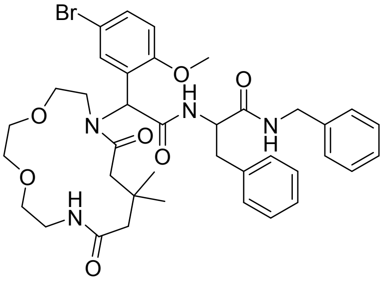

^{1}H NMR (500 MHz, Chloroform-d, 1:1 diastereomeric ratio) δ 8.00 (m, 1H), 7.50 (t, J = 2.1 Hz, 2H), 7.48 (s, 1H), 7.44 (d, J = 2.5 Hz, 1H), 7.42 (d, J = 2.4 Hz, 1H), 7.33 – 7.29 (m, 3H), 7.28 – 7.25 (m, 4H), 7.25 – 7.14 (m, 10H), 7.12 (d, J = 7.5 Hz, 1H), 6.96 (d, J = 7.3 Hz, 1H), 6.82 (d, J = 8.7 Hz, 1H), 6.72 (d, J = 8.8 Hz, 2H), 6.59 (d, J = 7.8 Hz, 1H), 5.52 (s, 1H), 5.20 (s, 1H), 4.77 – 4.63 (m, 2H), 4.47 (dd, J = 5.8, 1.7 Hz, 2H), 3.79 (s, 4H), 3.68 (s, 6H), 3.50 (dtd, J = 15.9, 10.2, 3.4 Hz, 9H), 3.45 – 3.30 (m, 9H), 3.32 – 3.14 (m, 9H), 2.47 (d, J = 5.7 Hz, 3H), 2.38 (d, J = 22.3 Hz, 3H), 2.18 (d, J = 9.5 Hz, 2H), 2.11 (d, J = 11.4 Hz, 2H), 1.05 (s, 6H), 0.97 (d, J = 6.1 Hz, 6H). ^{13}C NMR (126 MHz, $CDCl_3$) δ 176.4, 172.8, 172.5, 171.4, 171.2, 169.9, 168.4, 156.7, 156.5, 138.6, 138.5, 138.3, 137.9, 133.6, 133.3, 133.2, 133.1, 132.7, 129.7, 129.5, 129.3, 129.1, 129.0, 128.9, 128.8, 128.7, 128.1, 128.1, 127.6, 127.5, 127.3, 127.1, 127.0, 126.3, 125.6, 114.0, 113.7, 112.8, 112.7, 69.8, 69.7, 69.6, 69.5, 67.9, 61.6, 59.7, 59.6, 59.3, 56.2, 56.1, 55.7, 47.9, 47.1, 43.9, 43.5, 42.2, 41.8, 39.5, 39.4, 36.6, 35.9, 34.6, 34.4, 34.3, 34.1, 29.9, 29.8.

SFC-MS data for compounds 6-8:

6

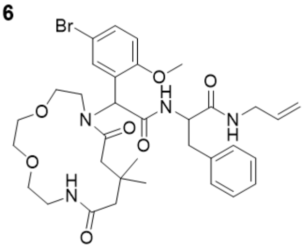

Chemical Formula: $C_{34}H_{45}BrN_4O_7$
Exact Mass: 700.25

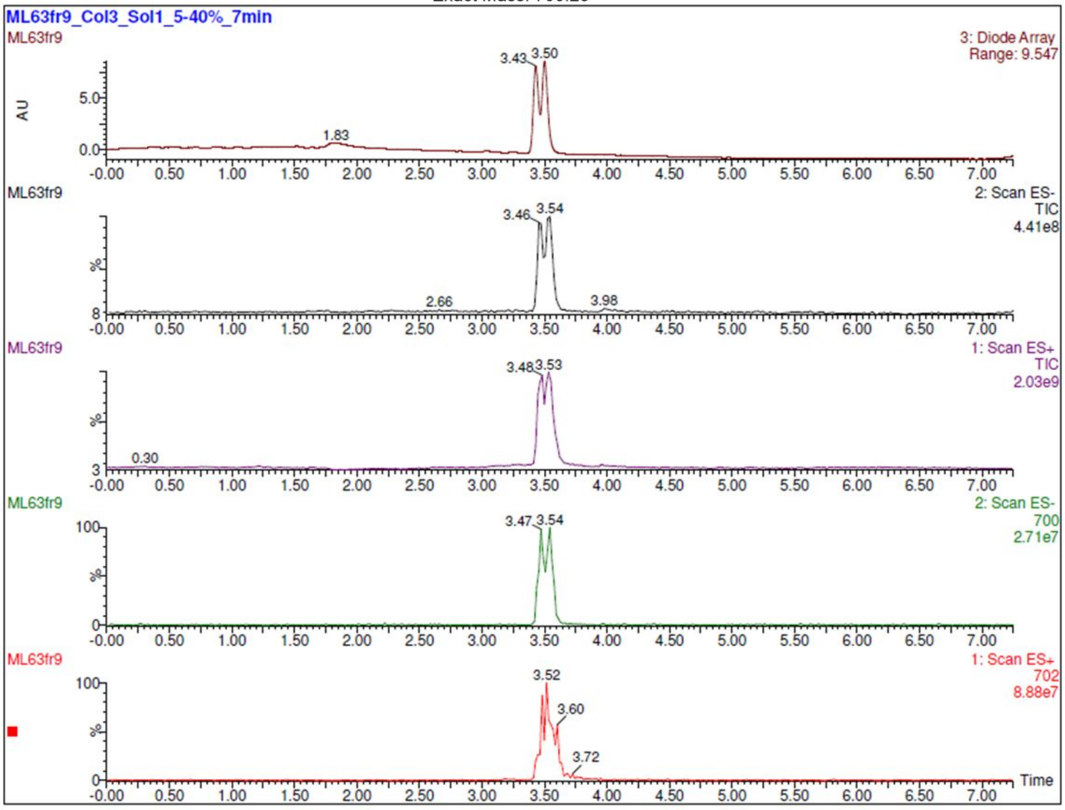

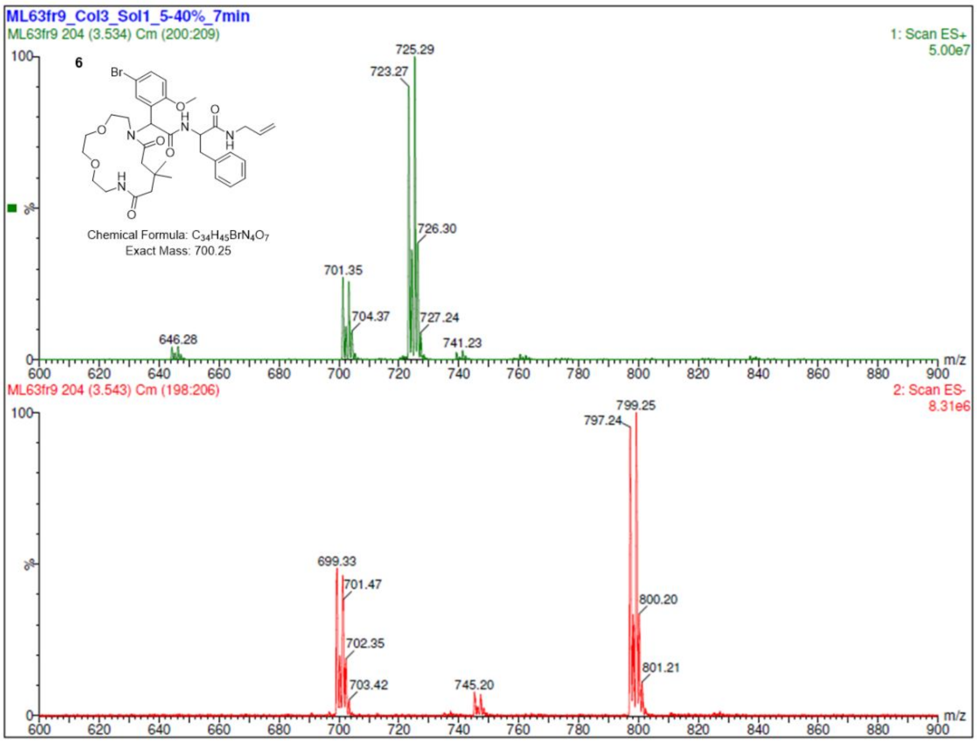

7

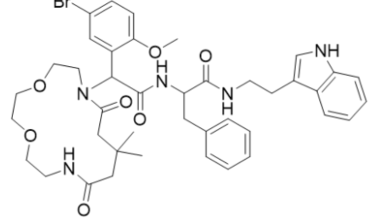

Chemical Formula: $C_{41}H_{50}BrN_5O_7$
Exact Mass: 803.29

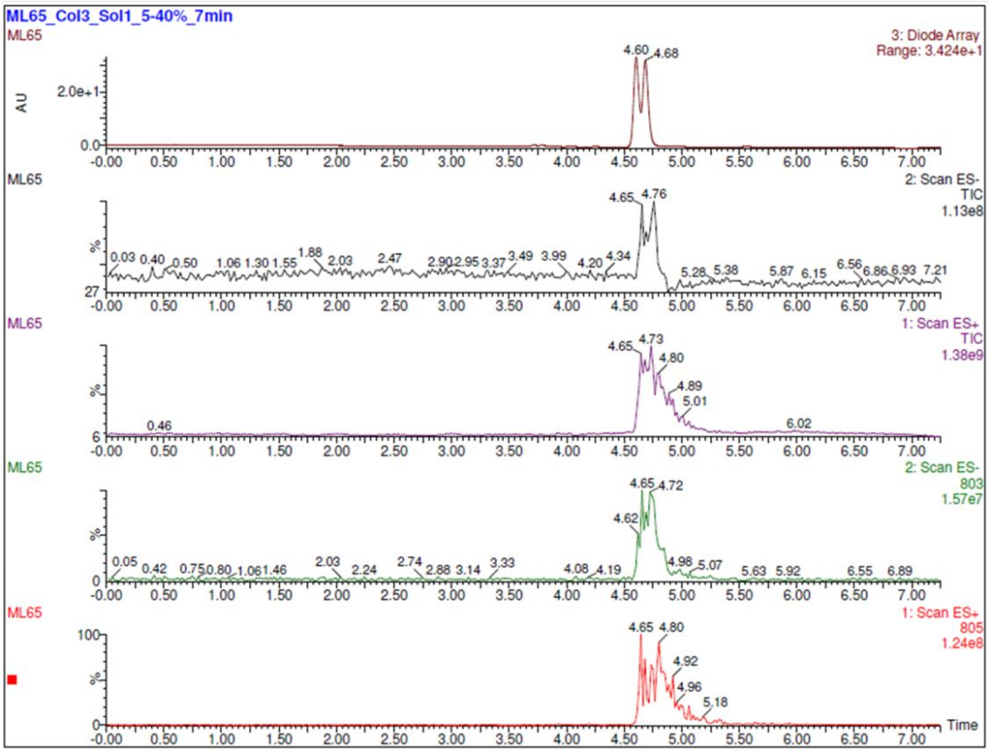

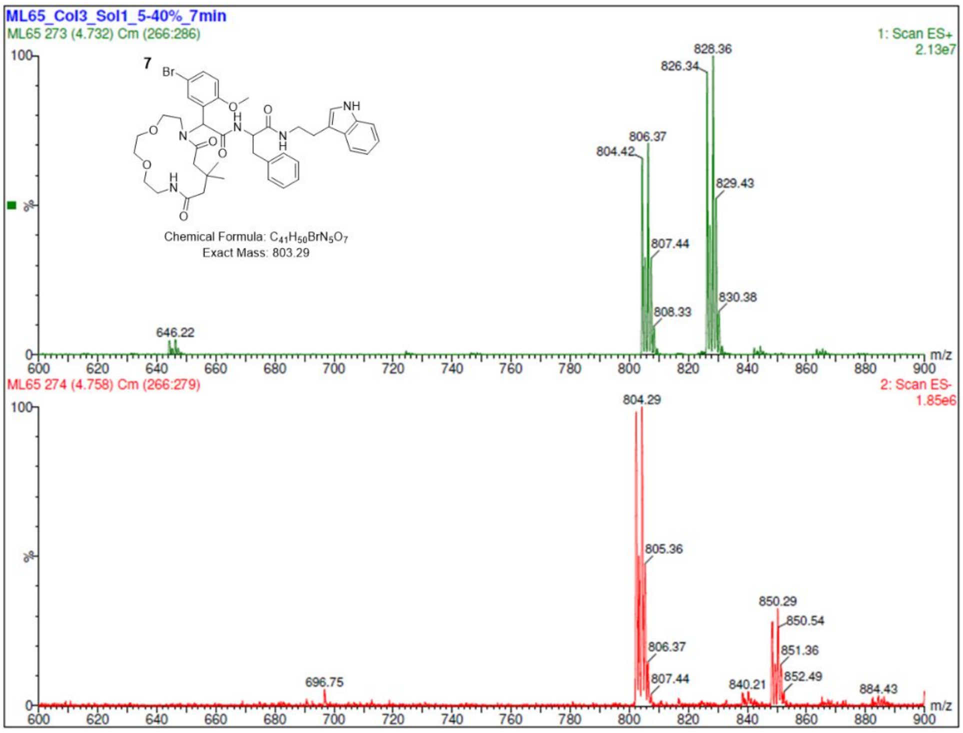

8

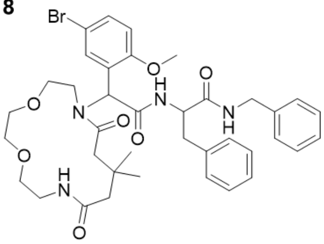

Chemical Formula: $C_{38}H_{47}BrN_4O_7$

Exact Mass: 750.26

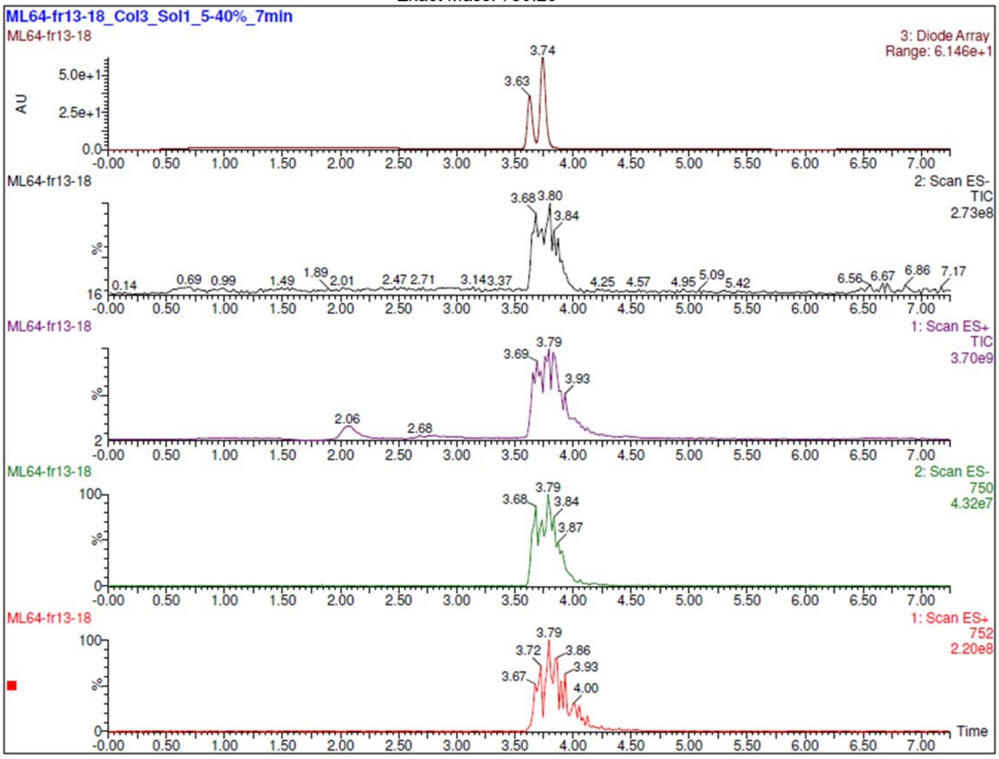

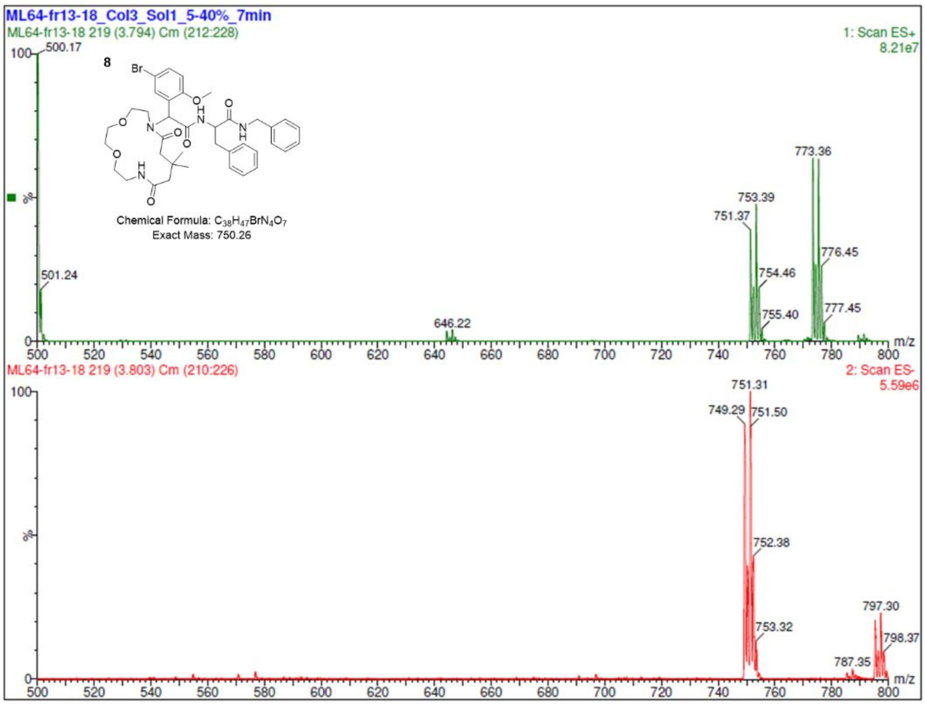

Figure SI-1. SFC-MS data for compounds 6-8.

SFC-MS separation for a diastereomeric mixture 6:

The optimal SFC-MS conditions were obtained eluting with the CO_2/MeOH mobile phase (83:17, v/v), 15.0 mL/min flow rate, 212.4 bar, and 40 °C oven temperature using a Chiralpak IC chiral column 10.0×250mm. It was observed that the product synthesized shows a diastereomeric mixture at the retention time of 37.80, 40.53, 51.53, and 60.39 minutes. Where separated product shows for 6-AB at the retention time of 33.06 and 35.02 minutes, 6-C at 42.57 minutes, and 6-D at 47.38 minutes (Fig. S2).

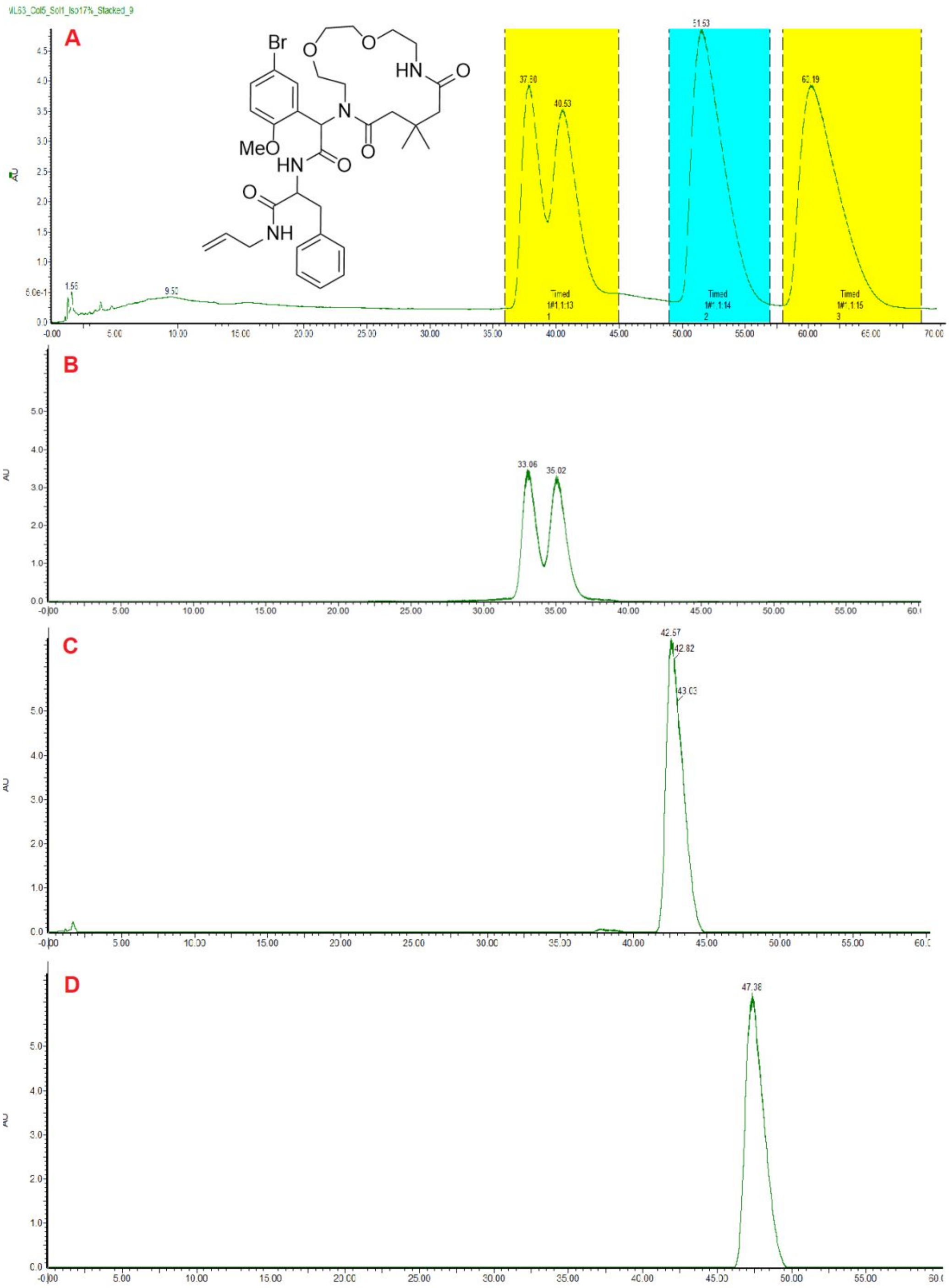

Figure SI-2. SFC-MS chromatograms of the separation of 6 on a Chiralpak IC chiral column: A, SFC-MS chromatogram of (rac)-6; B, SFC-MS chromatogram of 6-AB; C, SFC-MS chromatogram of 6-C; D, SFC-MS chromatogram of 6-D.

HRMS data for the separated compounds 6AB, 6C and 6D:

High resolution mass spectra for the rac mixture and separated compounds were measured with mass error < 5ppm using short LC gradient on C18 column, from Up+FA to ACN+FA.
Averaged extracted ions in retention time window 5.01-5.19 min, covering the peak with RT 5.09 in the TIC, in the mass range 700-707 m/z giving the expected molecular formula. Theoretical mass spectrum, calculated upon predicted molecular formula. Typical signature of Bromine containing compound.

HRMS for rac 6:

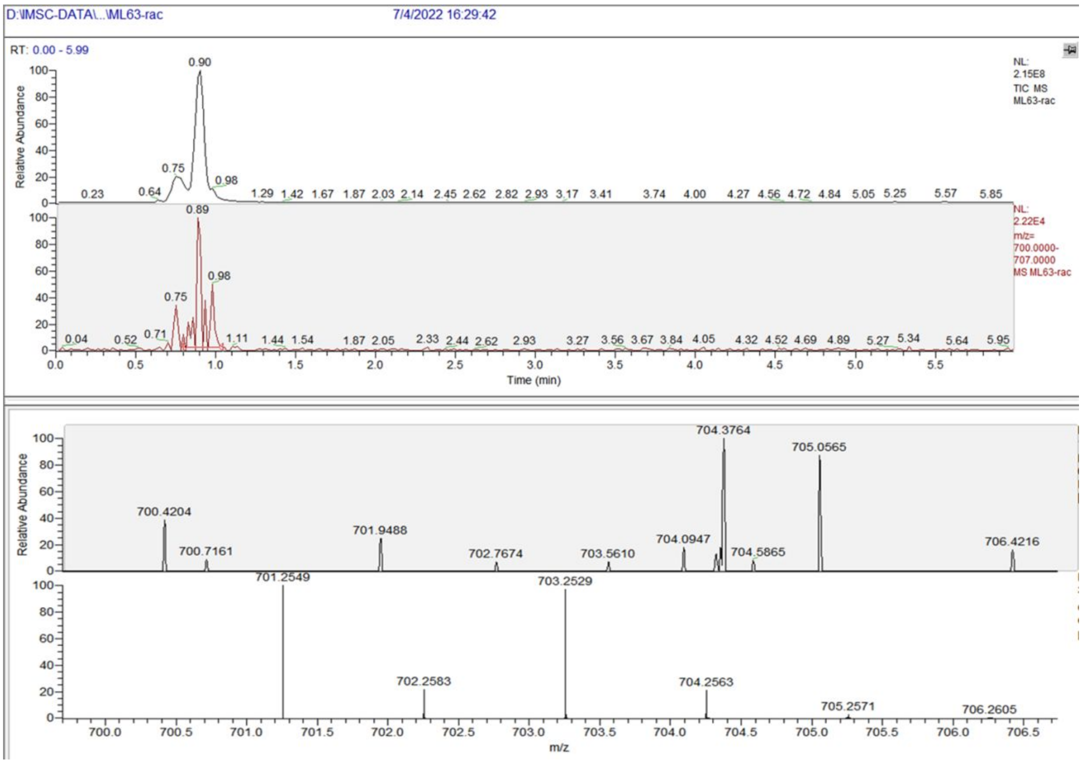

HRMS for cpmpound 6AB:

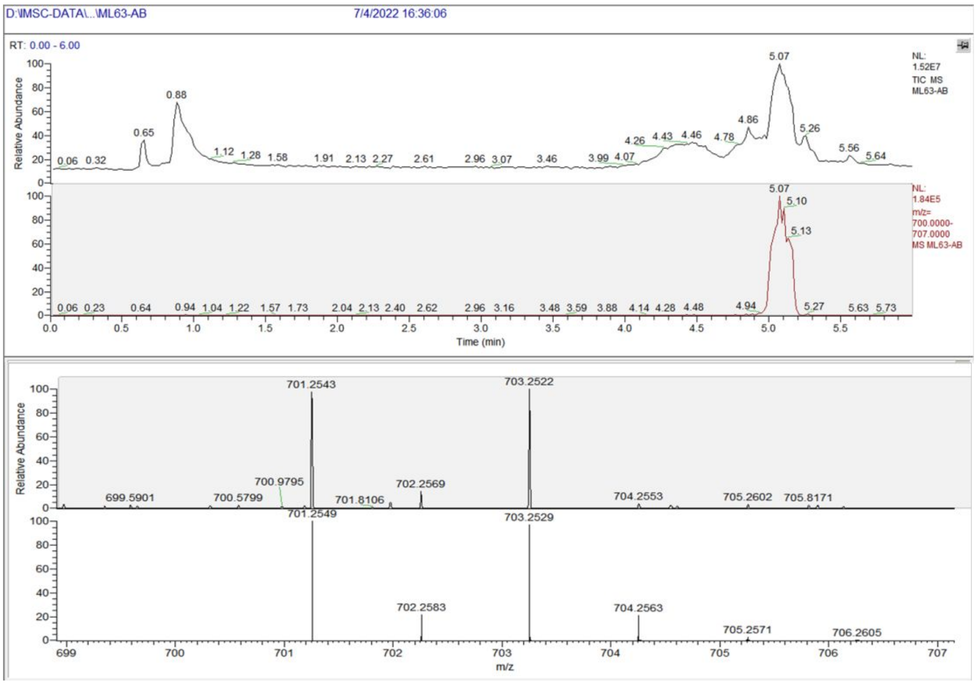

HRMS for cpmpound 6C:

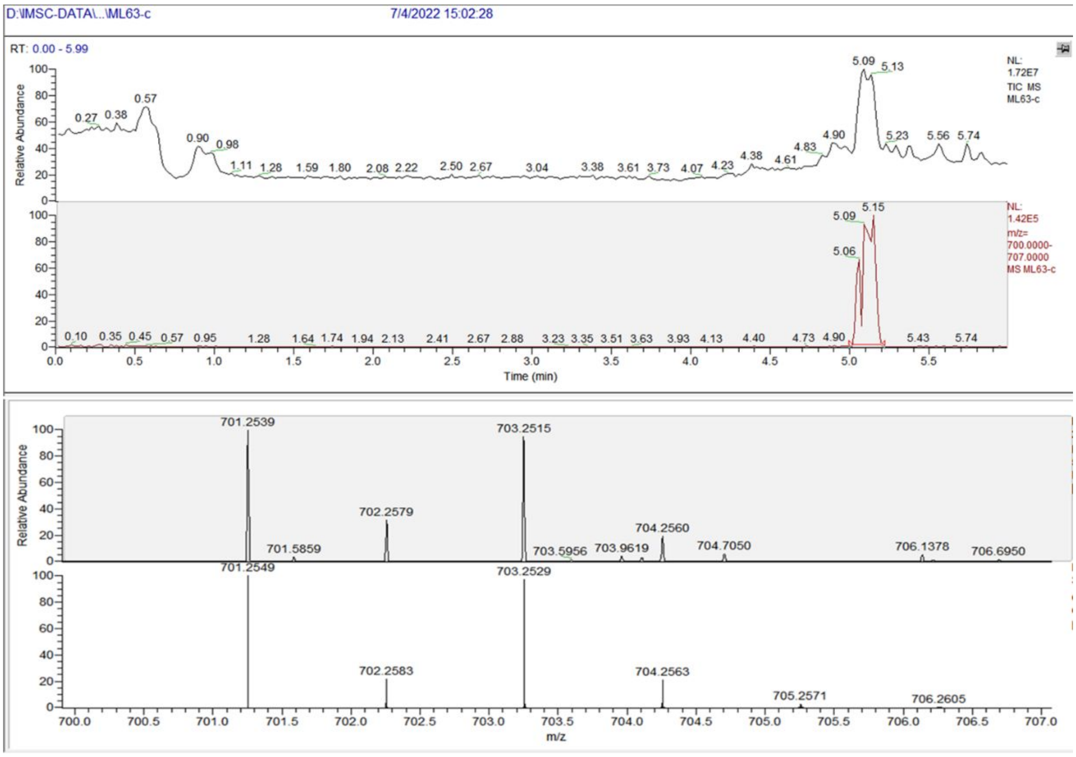

HRMS for cpmpound 6D:

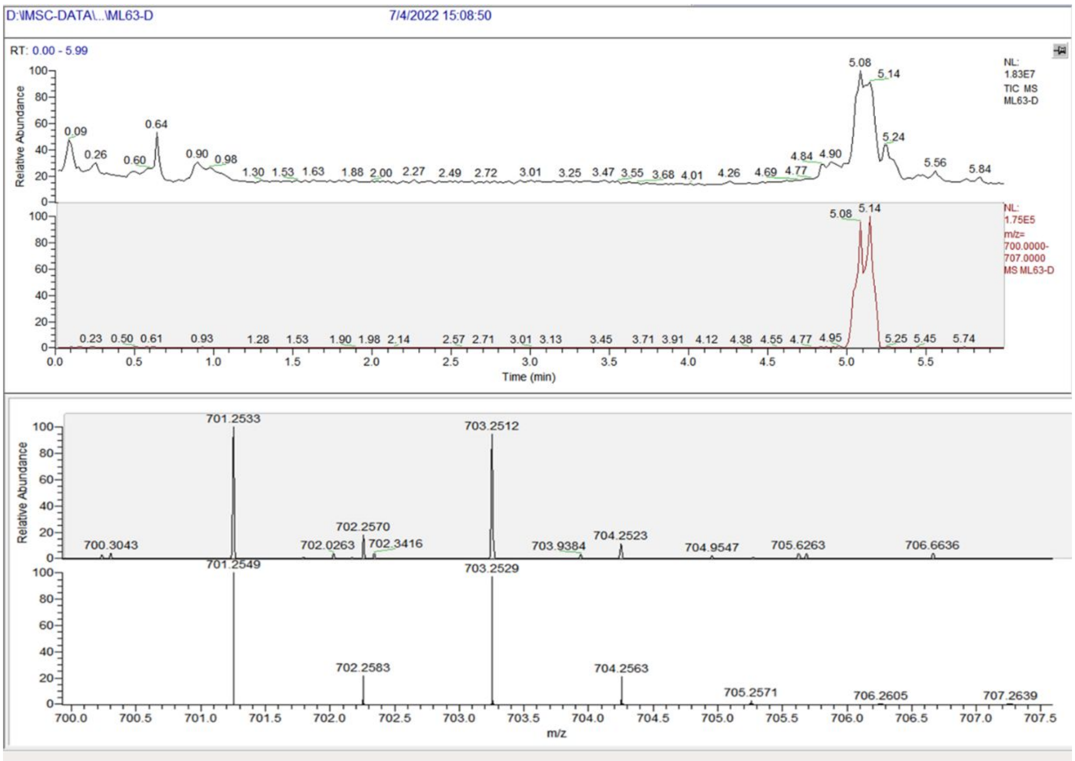

Figure SI-3. HRMS data for compounds 6rac, 6AB, 6C and 6D.

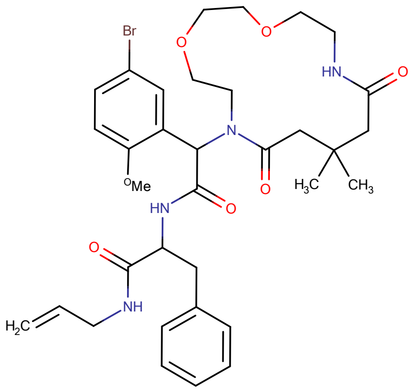

^{1}HNMR and ^{13}CNMR data for the separated compounds 6AB, 6C and 6D:

N-allyl-2-(2-(5-bromo-2-methoxyphenyl)-2-(10,10-dimethyl-8,12-dioxo-1,4-dioxa-7,13-diazacyclopentadecan-7-yl)acetamido)-3-phenylpropanamide 6AB:

^{1}H NMR (500 MHz, $CDCl_3$) δ 7.47 (d, J = 2.5 Hz, 1H), 7.42 (dd, J = 8.7, 2.5

Hz, 1H), 7.29 – 7.23 (m, 2H), 7.18 (t, J = 7.5 Hz, 3H), 6.99 (t, J = 5.7 Hz, 1H), 6.89 (t, J = 6.0 Hz, 1H), 6.75 (d, J = 8.8 Hz, 1H), 6.53 (d, J = 8.0 Hz, 1H), 5.86 – 5.76 (m, 1H), 5.52 (s, 1H), 5.18 – 5.07 (m, 2H), 4.65 (q, J = 7.5 Hz, 1H), 3.88 (t, J = 5.9 Hz, 2H), 3.75 (s, 3H), 3.60 – 3.16 (m, 14H), 2.59 – 2.43 (m, 4H), 1.07 (d, 6H) ppm. **^{13}C NMR** (126 MHz, $CDCl_3$) δ 176.1, 172.5, 170.9, 169.6, 156.9, 156.4, 137.6, 133.8, 133.0, 132.7, 129.1, 128.6, 126.7, 125.2, 116.4, 113.4, 112.3, 69.6, 69.3, 69.2, 67.7, 59.2, 55.9, 55.2, 47.5, 46.9, 42.1, 41.9, 39.1, 36.0, 34.0, 29.7, 29.5 ppm.

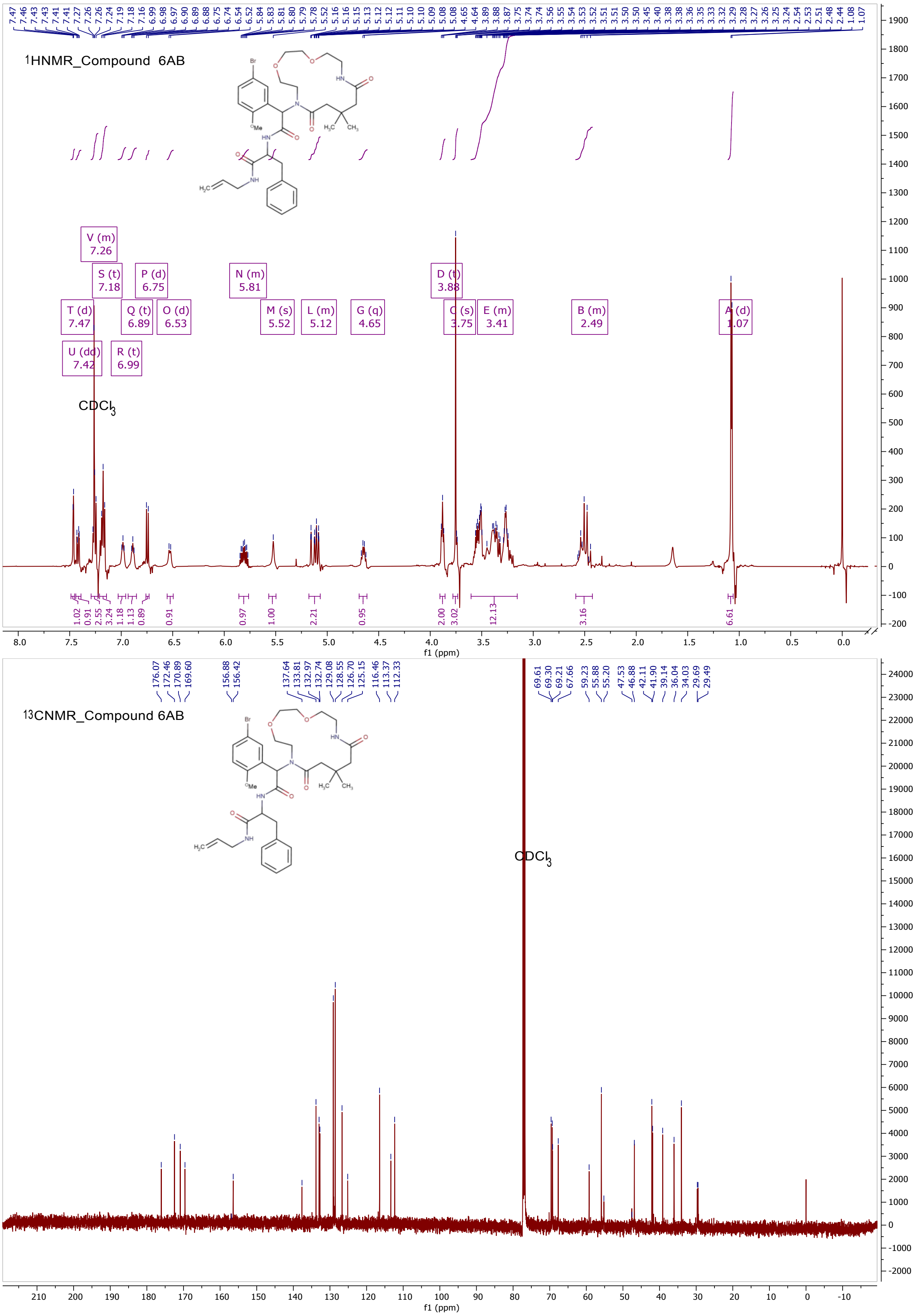

Figure SI-4: ^{1}HNMR and ^{13}CNMR for compound 6AB.

N-allyl-2-(2-(5-bromo-2-methoxyphenyl)-2-(10,10-dimethyl-8,12-dioxo-1,4-dioxa-7,13-diazacyclopentadecan-7-yl)acetamido)-3-phenylpropanamide 6C:

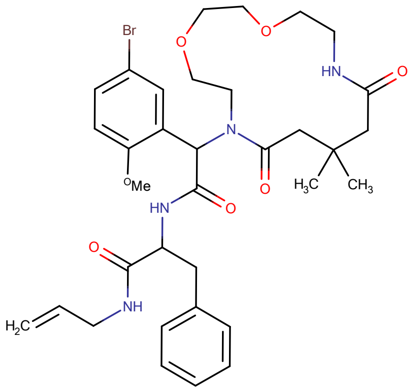

^{1}H NMR (500 MHz, $CDCl_3$) δ 7.56 (t, 1H), 7.49 – 7.43 (m, 2H), 7.22 – 7.15 (m, 3H), 6.98 (dd, J = 6.3, 2.9 Hz, 2H), 6.78 (d, J = 8.6 Hz, 1H), 6.68 (t, J = 6.2 Hz, 1H), 6.04 (s, 1H), 5.87 – 5.72 (m, 1H), 5.20 (s, 1H), 5.10 – 4.98 (m, 2H), 4.64 (s, 1H), 4.12 – 4.03 (m, 1H), 3.74 (s, 3H), 3.75 – 3.18 (m, 14H), 3.09 (dd, J = 14.3, 4.9 Hz, 1H), 2.65 – 2.39 (m, 4H), 1.06 (d, J = 10.8 Hz, 6H) ppm. **^{13}C NMR** (126 MHz, $CDCl_3$) δ 176.1, 172.3, 170.7, 168.2, 156.1, 137.1, 134.0, 132.9, 132.4, 129.0, 128.6, 126.7, 125.8, 115.6, 113.6, 112.3, 69.6, 69.4, 69.3, 67.8, 61.4, 55.8, 54.8, 51.0, 47.1, 41.8, 41.7, 39.4, 36.1, 34.0, 29.8, 29.5 ppm.

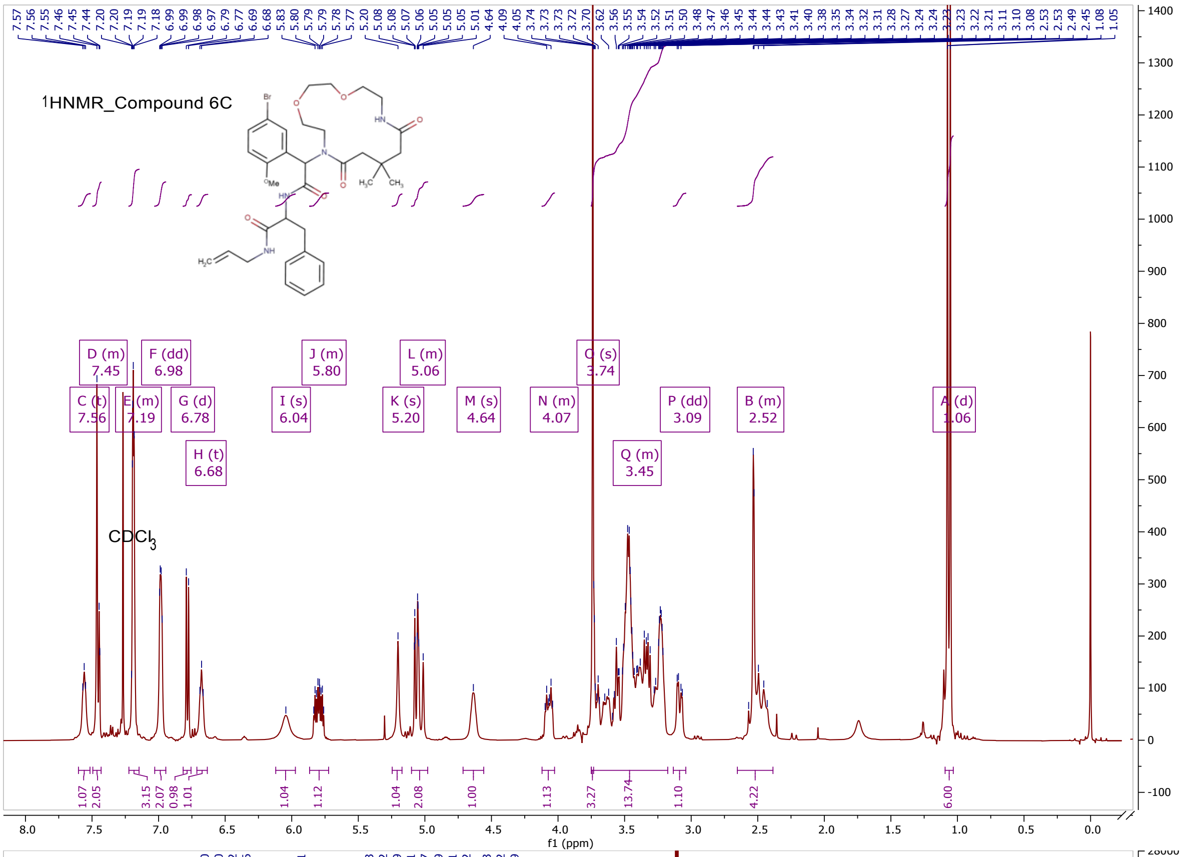

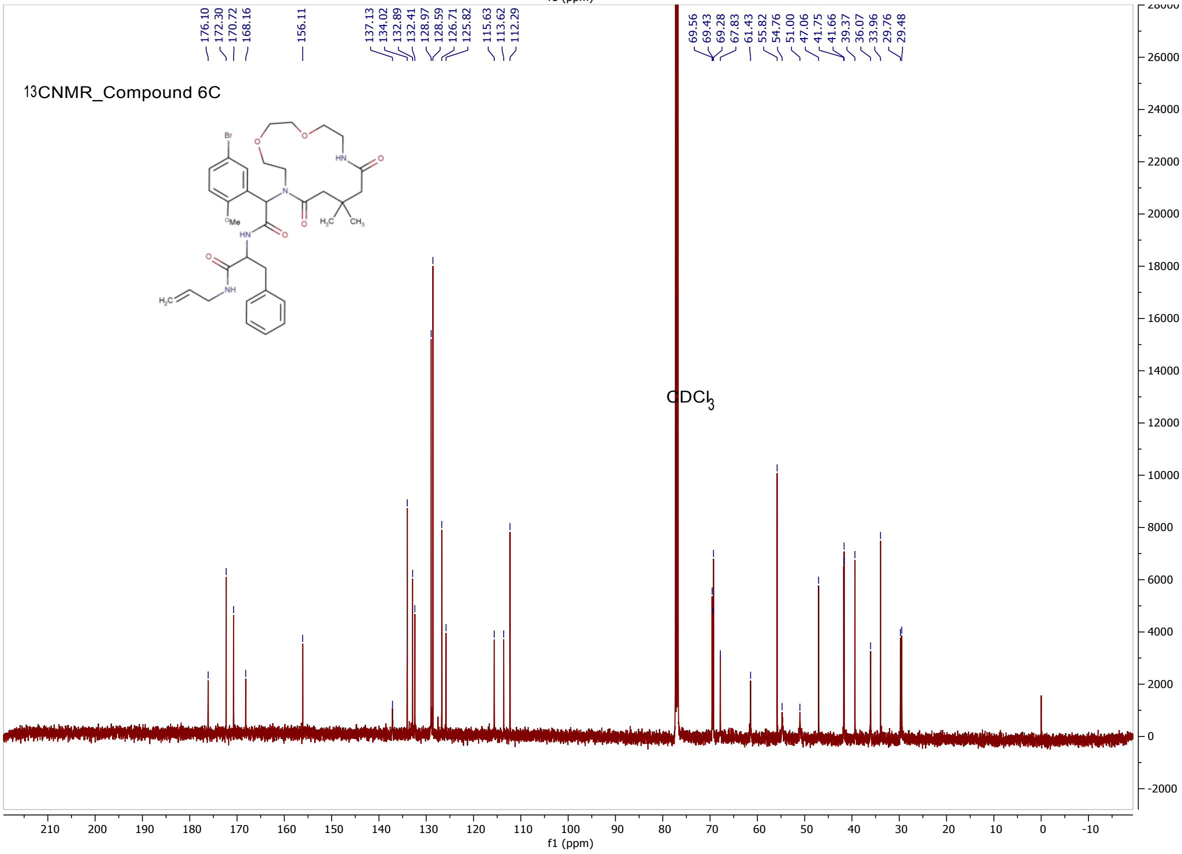

Figure SI-5: ^{1}HNMR and ^{13}CNMR for compound 6C.

N-allyl-2-(2-(5-bromo-2-methoxyphenyl)-2-(10,10-dimethyl-8,12-dioxo-1,4-dioxa-7,13-diazacyclopentadecan-7-yl)acetamido)-3-phenylpropanamide (6D)

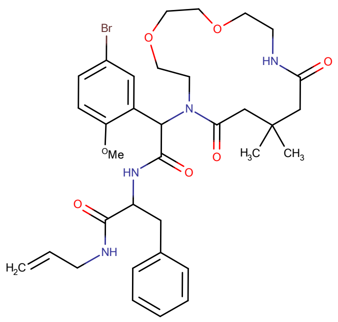

^{1}H NMR (500 MHz, $CDCl_3$) δ 7.55 (s, 1H), 7.49 – 7.43 (m, 2H), 7.21 – 7.16 (m, 3H), 7.01 – 6.95 (m, 2H), 6.78 (d, J = 8.6 Hz, 1H), 6.67 (s, 1H), 6.03 (s, 1H), 5.80 (ddt, J = 17.2, 10.3, 5.0 Hz, 1H), 5.20 (s, 1H), 5.10 – 4.99 (m, 2H), 4.63 (s, 1H), 4.07 (ddd, J = 16.1, 6.8, 5.0 Hz, 1H), 3.74 (s, 3H), 3.71 – 3.17 (m, 14H), 3.08 (dd, J = 14.0, 4.9 Hz, 1H), 2.59 – 2.39 (m, 4H), 1.06 (d, J = 11.2 Hz, 6H) ppm. **^{13}C NMR** (126 MHz, $CDCl_3$) δ 176.1, 172.3, 170.7, 168.2, 156.1, 137.2, 134.0, 132.9, 132.4, 129.0, 128.6, 126.7, 125.8, 115.6, 113.6, 112.3, 69.6, 69.4, 69.3, 67.8, 61.5, 55.8, 54.7, 51.1, 47.1, 41.7, 41.7, 39.4, 36.1, 34.0, 29.8, 29.5 ppm.

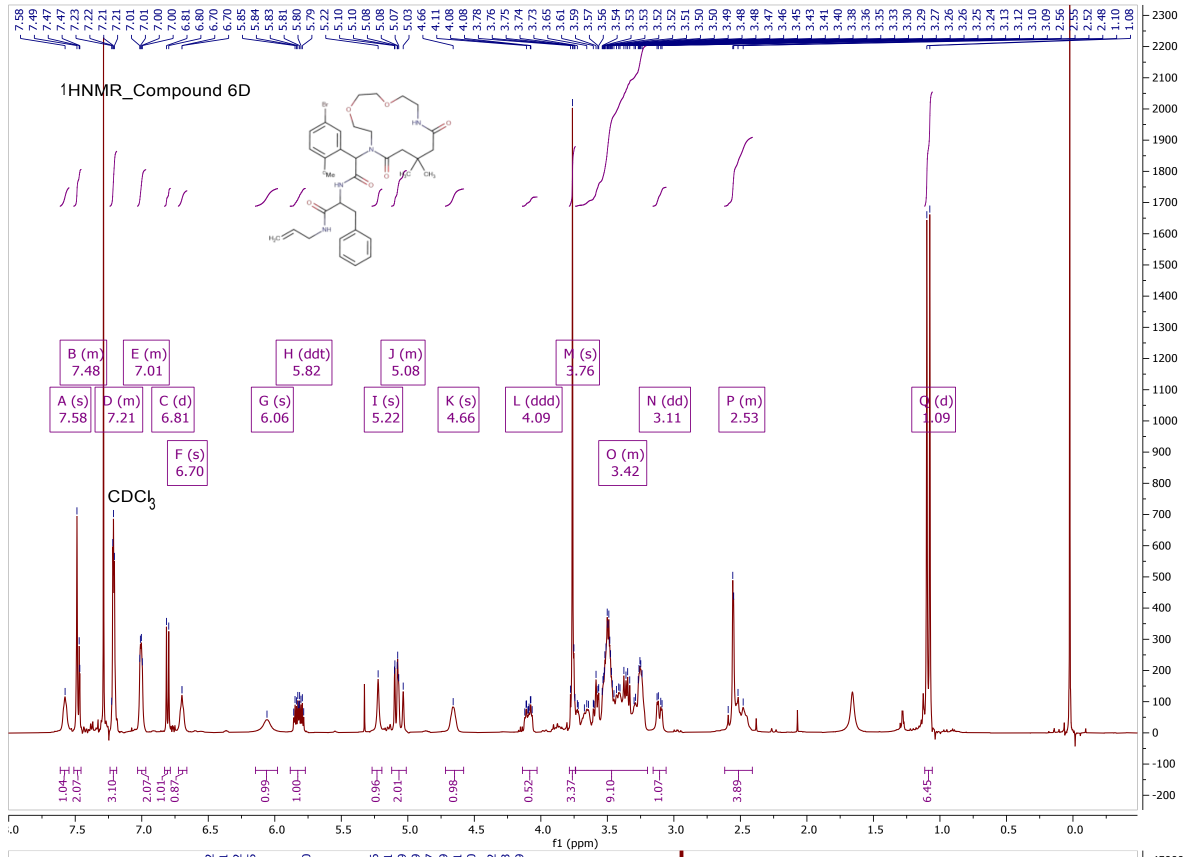

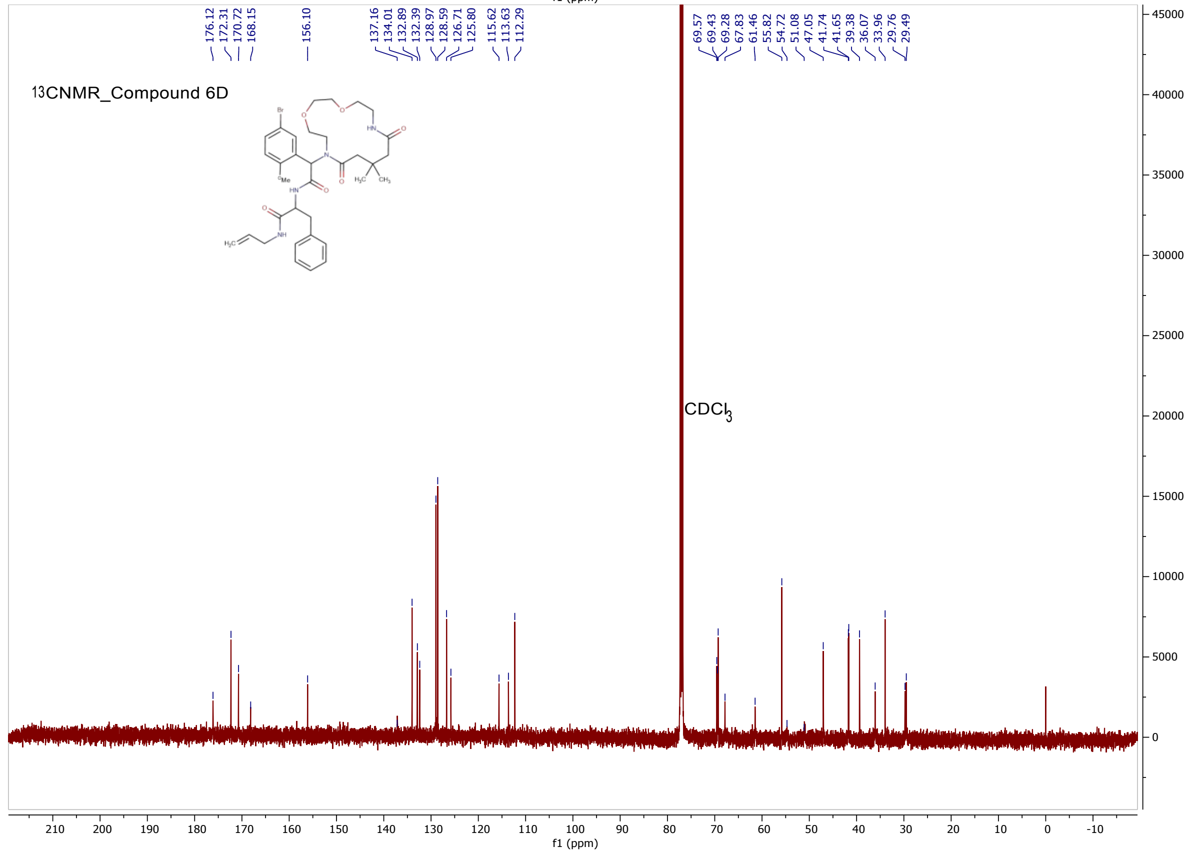

Figure SI-6: ^{1}HNMR and ^{13}CNMR for compound 6D.

Evaluation of inhibitory activity of compounds towards IL17:

Biophysical Assay:

IL-17a protein was produced following the protocol published in [PMID: 28839267]. Briefly, the protein is refolded from inclusion bodies by shock dilution into Tris-Base huffer and then purified by hydrophobic interaction chromatography and size exclusion chromatography. Protein was eluted from SEC in MES buffer and concentrated to 0.59 mg/ml prior to storage at -80°C.

MST measurements were performed on a Monolith NT.115 instrument (Nanotemper Technologies, GmbH). Purified IL-17A sample was labeled with the Monolith His-Tag Labeling Kit RED-tris-NTA (MO-L008) according to the supplied protocol (Nanotemper Technologies, GmbH). All measurements were performed in triplicate in PBS buffer (including 0.05% Tween) using standard capillaries (K002, Nanotemper Technologies, GmbH). Labeled IL-17a was used at a final concentration of 50 nM according to the manufacturer's recommendations. All measurements were performed at 20%-100% LED excitation power and 40 % MST power. Compounds were titrated in 1:1 dilution starting at 250 μM-1000 μM. All binding reactions were incubated for 30 min at room temperature followed by centrifugation at 10,000 x g before loading into the capillaries.

Table SI-1: MST screening result for compound 6, 7, 8, 6AB, 6C, 6D.

Compound	MST curves (Data & fit)	Kd
6	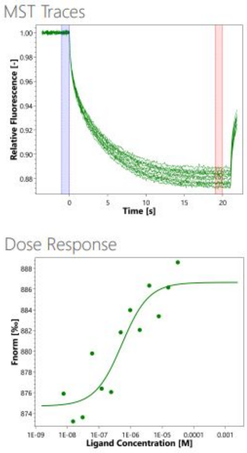	507 nM
7	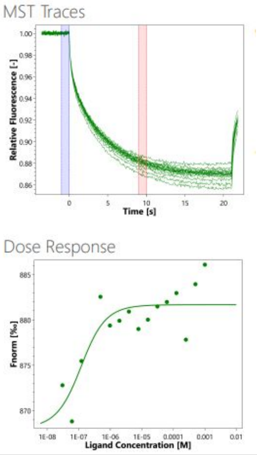	94 μM
8	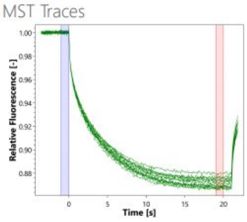	51.1μM

	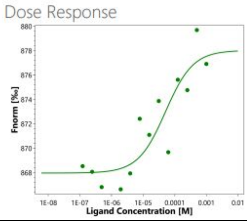	
6AB	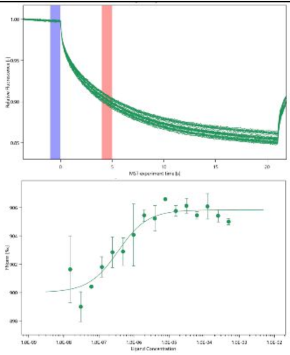	328 nM
6C	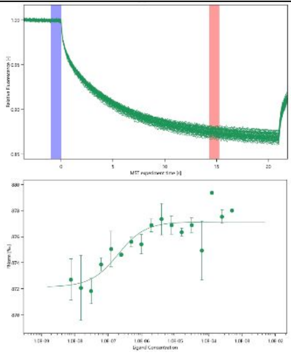	170 nM
6D	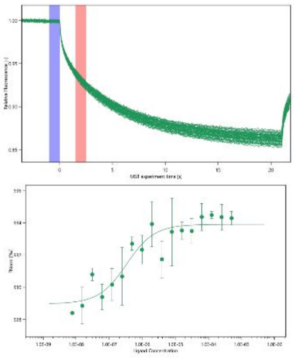	309 nM

Docking procedure, library construction, graphs and pictures of pharmacophore:

Molecular modeling

The crystal structure of IL-17A in complex with macrocycle inhibitor was fetched from the protein data bank (PDB) under the accession code 5HI4 and treated as receptor. Before modeling our virtual library of compounds, the receptor was prepared by removing crystallographic water molecules and the chains of CAT-2000 FAB antibody. Next, we prepared twenty-two virtual

combinatorial libraries containing 480 macrocycles each based on our in-house building block availability. Reactor was used for enumeration and reaction modeling (http://www.chemaxon.com). Ugi multicomponent reaction was employed as a template. This resulted in a total of 12000 molecules Unique SMILES strings can be found in the macrocycle_library.xlsx. We studied three structural variables: firstly, the ring size (e.g., from 9 to sixteen), secondly, the presence of heavy atoms such as sulfur and/or oxygen and finally lipophilic functional groups (e.g., dimethyl, cyclopentyl, cyclohexyl). All possible conformations and stereoisomers were generated with Mcnf module in Moloc (http://www.moloc.ch/). At this stage, no conformational constrains were specified. Next, the structures were then anchored with Openbabel[4] to the bis-amide moiety of the co-crystalized inhibitor for optimal interaction with the amide backbone of Leu-97 of chains A/B. The command used is "obfit *smartcode* anchor.sdf macrocycle_library.sdf > anchored.sdf" where *smartcode* corresponds to the SMARTs string of the anchor generated with Maestro (Schrödinger Release 2016-3: Maestro, Schrödinger, LLC, New York, NY, 2016). We then pool down the number of possible candidates with Pharmit [5] by applying a four-points pharmacophore and the exclusion shape criteria. The pharmacophore consists of two hydrogen bond acceptor features applied to the two amide oxygen atoms and two hydrogen bond donor features given by the amide nitrogen. A tolerance of 1.00 was applied to the exclusion volume around the receptor surface to exclude conformer with steric clashes. Candidates were energy minimized with Mold3d in Moloc. Finally, the poses were visually inspected with Pymol (The PyMOL Molecular Graphics System, Version 2.0 Schrödinger, LLC.) while cooperativity binding scores and protein/ligand interactions were predicted with Scorpion [6].

Docking of Macrocycle 6 Stereoisomers – Methodology

The 3D structure of **6** was done with Mol3d in Moloc (http://www.moloc.ch/), after which we manually assigned the desired stereochemistry to the two chiral carbons using the "invert" function in Pymol (https://pymolwiki.org/index.php/Invert). The conformations of the four stereoisomers were obtained using the RING-SHAPE algorithm incorporated in the Mcnf module we recently benchmarked (DOI: 10.1021/acs.jcim.0c01038) with the constraint of maintaining the chirality at the atoms. The batch command for this step is "Mcnf -w0 -e20 -c2". We anchored the conformations to the reference bis-amide with obfit in Openbabel [4] and then filtered them with Pharmit[5] to remove those clashing with IL-17A receptor surface. The resulting hits were then minimized with Mol3d in the receptor cavity using the batch command "Mol3d -e rec.mab -w0.01". Finally, both the cooperativity binding network scores and interactions were enumerated, parsed and classified in an automated fashion using a tailored python script.

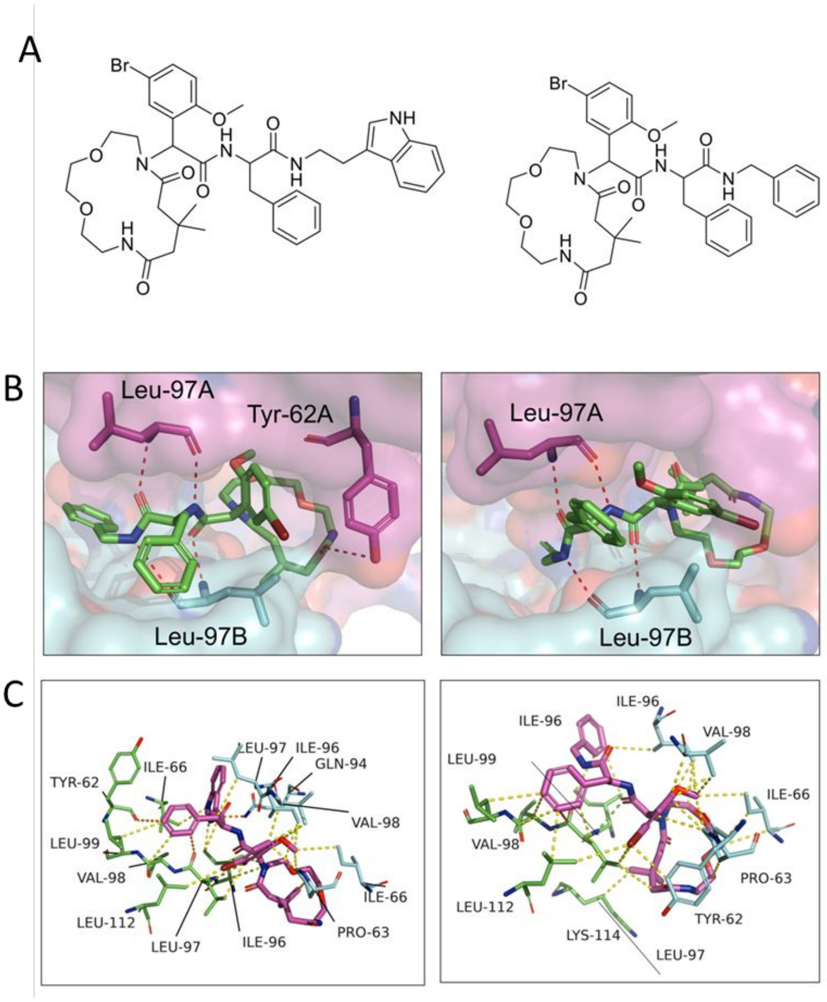

Figure SI-7: Docking poses of **7** and **8** in IL-17A interface. A) The 2D chemical structure of compound 7 and 8; B) The receptor cavity is represented as surface while the macrocycles bonds as green sticks. Monomers B and C are colored in cyan and magenta respectively. C) Displays the dense network of Van der Waals and few pi-pi interactions as yellow and orange dotted lines.

Table SI-2: Interaction network summary of three macrocycle hits. H-bond: hydrogen bonds, hbon_pi: hydrogen bond – pi, vdW: Van der Waals, poor_ang: interactions with non-ideal bond angle; unfav: interactions potentially unfavorable (e.g., surface clash); unclass: unclassified interaction.

Cooperativity binding network Parameter	Comp. 6 (SS)	Comp. 7	Comp.8
**hbond**	5	5	5
**hdon_pi**	0	1	0
**vdW**	22	28	20
**pi_pi**	4	2	3
**poor_ang**	2	6	4
**unfav**	1	2	4
**unclass**	0	3	5

**Scorpion Score**	14.6	19.3	12.4

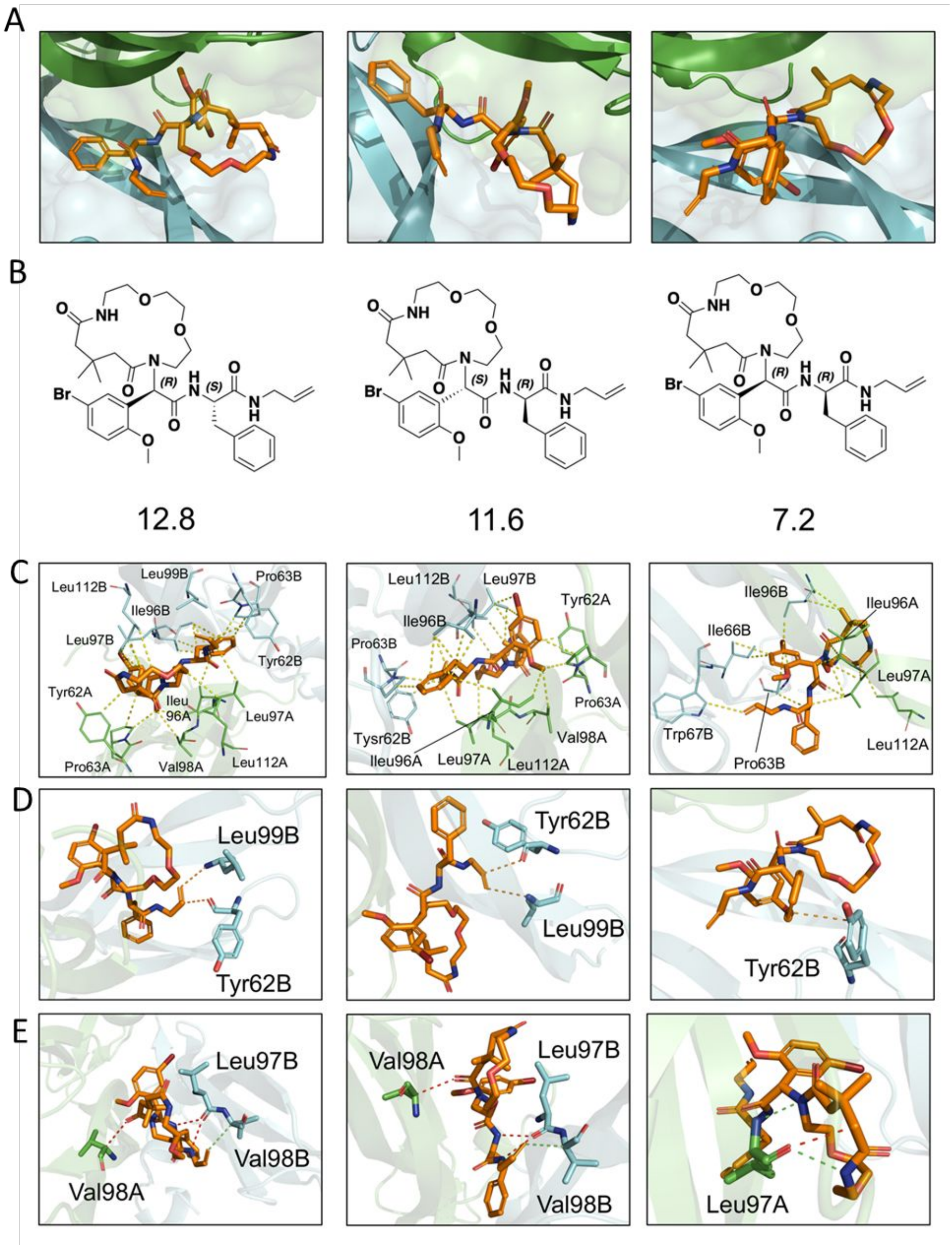

Figure SI-8: Panel of the three stereoisomers of macrocycle **6**: A) RS (left column), SR (middle column) and RR (right column). The A and B chains of IL-17A are depicted as green and cyan ribbons, respectively. The same color pattern is applied to amino acids (stick representation) involved in molecular contacts with **6**. B) The 2D chemical structure is shown the score as predicted by Scorpion. Second panel row; C) Van der Waals interactions (yellow dotted lines). The atomic radius of the amino acids has been rescaled for better visualization. D) Third row: pi – pi interactions (orange dotted lines). Fourth row; E) hydrogen bond (red dotted lines) and hydrogen bond – pi interactions (green dotted lines).

References:

1.Doemling, A.; Madhavachary, R.; Abdelraheem, E. M. M.; Rossetti, A.; Twarda-Clapa, A.; Musielak, B.; Kurpiewska, K.; TBv, K.; Holak, T. A., Two Steps towards Complex and Artificial Medium- and Macrocycles. *Angewandte Chemie* **2017**.

2.Dömling, A.; Beck, B.; Fuchs, T.; Yazbak, A., Parallel synthesis of arrays of amino-acid-derived isocyanoamides useful as starting materials in IMCR. *Journal of combinatorial chemistry* **2006,** *8* (6), 872-880.

3.Sabot, C.; Kumar, K. A.; Meunier, S.; Mioskowski, C., A convenient aminolysis of esters catalyzed by 1, 5, 7-triazabicyclo [4.4. 0] dec-5-ene (TBD) under solvent-free conditions. *Tetrahedron letters* **2007,** *48* (22), 3863-3866.

4.Hummell, N. A.; Revtovich, A. V.; Kirienko, N. V., Novel immune modulators enhance Caenorhabditis elegans resistance to multiple pathogens. *Msphere* **2021,** *6* (1), e00950-20.

5.Sunseri, J.; Koes, D. R., Pharmit: interactive exploration of chemical space. *Nucleic acids research* **2016,** *44* (W1), W442-W448.

6.Kuhn, B.; Fuchs, J. E.; Reutlinger, M.; Stahl, M.; Taylor, N. R., Rationalizing tight ligand binding through cooperative interaction networks. *Journal of chemical information and modeling* **2011,** *51* (12), 3180-3198.
